# Advancing pet care through technology: An evaluation of the LINE-based VET PROMPT notification system

**DOI:** 10.5455/javar.2025.l947

**Published:** 2025-09-08

**Authors:** Supaporn Somrup, Anucha Sirimalaisuwan, Warangkhana Chaisowwong, Kannika Na–Lampang

**Affiliations:** 1 Faculty of Veterinary Medicine, Chiang Mai University, Chiang Mai, Thailand; 2 Research Center for Veterinary Biosciences and Veterinary Public Health, Faculty of Veterinary Medicine, Chiang Mai University, Chiang Mai, Thailand

**Keywords:** Notification system, pets, pet healthcare, pet owners, pet online, pet online healthcare

## Abstract

**Objective:**

This study evaluated the effectiveness of the VET PROMPT notification, accessed via the LINE Application Official Account (LAOA).

**Materials and Methods:**

In enhancing communication between veterinarians and pet owners. By leveraging pets online, it offers a convenient and cost-effective solution for pet owners to receive timely and pertinent veterinary information, appointment notifications, and disease prevention guidance for their pets. The research involved administering a satisfaction questionnaire to pet owners who subscribed to the LAOA–VET PROMPT group and analyzing responses using statistical measures such as percentage, mean, standard deviation, and one-way ANOVA.

**Results:**

Findings revealed high levels of satisfaction among users, particularly with the system’s responsiveness and the completeness and accessibility of online pet healthcare. Furthermore, the study discovered significant variations in satisfaction levels based on the type of pet owned, indicating that the specific needs and preferences of different pet owner groups uniquely influence their satisfaction with the VET PROMPT.

**Conclusion:**

Overall, the integration of automatic notifications into pet owners’ mobile devices significantly enhanced the management and care of pets through streamlined communication.

## Introduction

In Thailand, the increasing trend of pet ownership reflects a broader integration of companion animals into family dynamics, recognized for significantly enhancing familial well-being and individual health [1]. Mueller et al. [2] substantiated that pet owners generally exhibit better health outcomes compared to non-pet owners, particularly among dog owners, with the observed health benefits largely stemming from the companionship pets provide. Moreover, communication plays a critical role in managing pet health care, with pet owners increasingly preferring online systems due to their ease, speed, and convenience, accommodating users across all age groups. This shift towards digital platforms is evident across a variety of activities, including transactions and social interactions through smartphones and applications such as Line and Facebook [3]. It was found that only a small proportion of pet owners currently receive appointment confirmations, despite a strong interest in receiving reminder notifications. Additionally, 53.8% of pet owners expressed a desire to receive medical updates more frequently than they presently do. While veterinarians predominantly rely on verbal communication, typically via phone calls, many pet owners actually prefer communication through text messages [4]. India has developed an online system that emphasizes digital interaction, incorporating messaging features and enabling pet owners to make direct inquiries online [5]. The accessibility of online systems is particularly advantageous for the elderly, enhancing their ability to receive information through mobile devices [6].

Furthermore, the adoption of telemedicine in veterinary care mirrors its rise in human medicine, offering significant cost savings by reducing the need for travel [7]. Collectively, these developments accentuate the importance of digital communication platforms in improving the accessibility and effectiveness of health management for pets and their owners, as well as in providing valuable information on blood donation in animals [8]. The growing adoption of veterinary telemedicine is providing pet owners with greater convenience by reducing the need for travel and offering easier access to consultations and appointment scheduling via mobile platforms. In addition, the implementation of online learning (e-learning) for veterinary telehealth education enables students and professionals to acquire knowledge and training remotely via digital platforms [9,10]. Video-based telemedicine has the potential to reduce stress levels and improve access to healthcare for cats when compared to traditional in-clinic visits [11,12]. It is recognized that while online systems offer significant benefits and convenience, their application remains subject to legal limitations and must be implemented in accordance with established medical ethics [13]. Moreover, to ensure reliable communication between veterinarians and animal owners, adherence to the principles outlined in the Telehealth Guidelines for Small Animal Practice is required [14–16].

In the field of veterinary medicine, leveraging online media as a notification tool is crucial for enhancing the dissemination of critical information to pet owners, particularly regarding disease prevention strategies. Javed et al. [17] highlight the urgent need for awareness about rabies and other lyssavirus-related diseases, which pose significant risks to both animals and humans and can lead to fatal outcomes if left unaddressed. Vaccinations, as recommended by the World Small Animal Veterinary Association’s Vaccination Guidelines Group (VGG), are essential preventive measures for dogs and cats [18]. The ambition to eradicate the rabies virus (RV) by 2030 underscores the challenges related to the accessibility of vital knowledge concerning the disease and post-­exposure vaccination protocols. Nujum et al. [19] identified pet animals as reservoirs for spreading methicillin-­resistant* Staphylococcus aureus* to human health [20], further stressing the importance of continuous education on ­animal disease prevention, which is critical not only for animal health but also for safeguarding human health. Such initiatives are imperative to maintaining the health of both pets and their owners, reinforcing the need for effective communication channels that can reach a broad audience.

The dissemination of knowledge about pet diseases, particularly viral infections in dogs and cats, has increasingly relied on online systems. During the COVID-19 pandemic, Applebaum et al. [21] surveyed 2,254 pet owners in the United States, identifying significant challenges in accessing veterinary care and obtaining guidance on preventing disease transmission from animals. This period highlighted the necessity for pet owners, many of whom were working from home, to access reliable veterinary information remotely. Concurrently, the integration of the Internet of Things (IoT) in veterinary care has expanded, as exemplified by the Novel Pet Collaborative Care (NPCC) system. This innovative approach utilizes IoT to enhance communication between pets and owners, improving the monitoring and management of pet health and activities, thereby promoting overall well-being [22]. In light of these developments, it is imperative to establish efficient channels that facilitate timely access to veterinary health information, enabling pet owners to effectively understand and implement disease control and prevention strategies both during public health crises and in normal conditions. Currently, appointment reminders for pet owners with veterinary clinic visits are delivered through two online digital platforms: email reminders and SMS reminders [23]*.* It was found that there was communication between the animal owner and the veterinarian by talking and making appointments directly via video [24].

In the field of veterinary medicine, leveraging online media as a notification tool is crucial for enhancing the dissemination of critical information to pet owners, particularly regarding disease prevention strategies. Singh et al. [17] highlight the urgent need for awareness about rabies and other lyssavirus-related diseases, which pose significant risks to both animals and humans and can lead to fatal outcomes if left unaddressed. Vaccinations, as recommended by the World Small Animal Veterinary Association’s VGG, are essential preventive measures for dogs and cats [18]. The ambition to eradicate the RV by 2030 underscores the challenges related to the accessibility of vital knowledge concerning the disease and post-­exposure vaccination protocols. Nujum et al. [19] identified pet animals as reservoirs for spreading methicillin-­resistant* S. aureus* to human health [20], further stressing the importance of continuous education on animal disease prevention, which is critical not only for animal health but also for safeguarding human health. Such initiatives are imperative to maintaining the health of both pets and their owners, reinforcing the need for effective communication channels that can reach a broad audience.

The dissemination of knowledge about pet diseases, particularly viral infections in dogs and cats, has increasingly relied on online systems. During the COVID-19 pandemic, Applebaum et al. [21] surveyed 2,254 pet owners in the United States, identifying significant challenges in accessing veterinary care and obtaining guidance on preventing disease transmission from animals. This period highlighted the necessity for pet owners, many of whom were working from home, to access reliable veterinary information remotely. Concurrently, the integration of the IoT in veterinary care has expanded, as exemplified by the NPCC system. This innovative approach utilizes IoT to enhance communication between pets and owners, improving the monitoring and management of pet health and activities, thereby promoting overall well-being [22]. In light of these developments, it is imperative to establish efficient channels that facilitate timely access to veterinary health information, enabling pet owners to effectively understand and implement disease control and prevention strategies both during public health crises and in normal conditions. Currently, appointment reminders for pet owners with veterinary clinic visits are delivered through two online digital platforms: email reminders and SMS reminders [23]*.* It was found that there was communication between the animal owner and the veterinarian by talking and making appointments directly via video [24].

The goal of this study is to significantly improve pet owners’ awareness and understanding of animal health issues through digital platforms by optimizing the use of online media channels for continuous and efficient dissemination of pet health information. By developing tailored media formats accessible via the LAOA, the study seeks to facilitate the effective transmission of crucial health information to pet owners. The ultimate objective is to ensure that pet owners receive timely and efficient updates, thereby maximizing the promptness and efficacy of information delivery through these online media channels.

## Materials and Methods

### Ethical approval

For this study, Ethical approval for the study was received from the Chiang Mai University Research Ethics Committee, which reviewed and approved the above research protocol based on international guidelines for human research protection, including the Declaration of Helsinki, the International Conference on Harmonization in Good Clinical Practice (ICH–GCP), and the Belmont Report. Date of approval was on 14 February 2023, Review method by full board, CMUREC Approval No. HS1/2566.

### Study outline

This study applied a non-probability purposive sampling method to select 400 pet owners, then added them to the LAOA group for data collection. This group consisted of pet owners with dogs or cats who received notifications via the VET PROMPT system. The study included only those who received notifications and responded to an online questionnaire in the sample. Out of the initial 400 participants, 147 completed the questionnaire, providing the necessary data for analysis. The remaining 253 participants were still pending receipt of their notifications. The study determined the sample size of 147 using the Yamane [25] formula, which facilitated the administration of a satisfaction questionnaire to effectively gather and analyze data from the participants who actively engaged with the notification system.

### Methods

In this study, participants were recruited through a digital process where they scanned a QR code to access a Google Form questionnaire and provided their consent. The questionnaire was structured into two sections: The first collected personal information, and the second evaluated participants’ satisfaction with the VET PROMPT system. Moreover, participants were required to scan another QR code to join a LINE group, which served as a platform for disseminating pet health notifications related to scheduled services such as combined vaccines, rabies vaccinations, flea and tick protection, and deworming. These notifications allowed pet owners to remain updated on their upcoming appointments. Following the receipt of these notifications, participants were asked to scan a QR code once more to record their satisfaction levels with the VET PROMPT system’s functionality. The Human Research Ethics Committee (Approval No. HS1/2566) approved the ethical guidelines for the research protocols, which included the collection of pet health information and participant satisfaction data.

### Study area and design

The samples were collected from owners of dogs and cats residing in Thailand. The population studied comprised individuals aged 18 years and older who owned at least one pet (a dog or cat) and lived in Thailand. This study employed a quasi-experimental research design with a one-group posttest. The sample group was selected from a specific population based on inclusion criteria: individuals who owned at least one pet (dog or cat), were capable of listening, speaking, reading, and writing in Thai, possessed a smartphone, and could download applications. The population included those who had basic proficiency in using the LINE application. The inclusion criteria for selecting volunteers included individuals aged 18 years or older who consented to participate in the research, could read Thai, had basic proficiency in using LINE, owned a smartphone, and could download the LINE Application Official Account. Exclusion criteria for not accepting volunteers into the project were individuals under 18 years of age, those unable to read Thai, those lacking basic proficiency in Line usage, those not owning a smartphone, those unable to download applications, or those who withdrew from the research project during data collection.

Satisfaction was assessed using a questionnaire employing rating scales, which included both positive and negative statements for respondents to indicate their level of agreement or disagreement. The 5-point Likert Scale method was utilized. The questionnaire underwent a quality assessment using the Index of Congruence (IOC), involving presentations to three experts who reviewed and rated the content’s validity. Each item achieved an IOC value ranging from 0.50 to 1.0. Items with an IOC value of 0.50 or higher were considered to have content validity. Subsequently, the reliability of the questionnaire was assessed using Cronbach’s alpha coefficient. The questionnaire was administered once to the target group, and the scores were analyzed, yielding an alpha coefficient value of 0.70 or higher.

### The criteria for the interpretation of mean scores

This study established criteria to interpret the mean scores of the 5-point Likert scale. The criteria were determined using the following formula for calculating the class interval:

Class interval = (Upper limit–Lower limit)/Number of class intervals

= (5–1)/5

= 0.80

The scoring criteria are as follows:

4.21–5.00: Very satisfied

3.41–4.20: Satisfied

2.61–3.40: Neutral

1.81–2.60: Dissatisfied

1.00–1.80: Very dissatisfied

### Statistical analysis

The data analysis for this research was divided into 2 parts: Descriptive analysis and inferential analysis. Statistical processing was conducted using R (4.4.1) and RStudio (421). The descriptive analysis entailed summarizing the data using frequency distributions, percentage values, frequency values, means, and standard deviations. For inferential analysis, Cronbach’s Alpha was used to analyze the reliability of each aspect of VET PROMT. Ordinal logistic regression was used to find the effect of pet type, age, education level, and salary as independent variables and satisfaction score as the dependent variable. All statistics are considered significant at *p* ≤ 0.05.

## Results

After collecting data from pet owners participating in the VET PROMPT system, all participants recorded vaccination, deworming, and parasite eradication information. Once the system processed this information, it promptly notified the pet owners to confirm the details. Additionally, the system would notify pet owners immediately when appointments with the veterinarian were due. Analysis of the demographic data collected from users of the VET PROMPT system revealed a predominance of male pet owners, who comprised 61.2 % of the sample (90 individuals), compared to female pet owners, who made up 38.8 % (57 individuals). Participant age ranges from 18 to 18–44 years old (Median = 20; Q1–Q3 = 19–21; IQR = 2). The age distribution among the pet owners indicated a majority of adolescents and adults, with adolescents aged 18–20 years comprising 53.06% of the respondents (78 individuals) and adults aged 21–60 years comprising 46.94% (69 individuals). The educational backgrounds of the pet owners varied, with those holding a bachelor’s degree representing the largest group at 68.7% (101 individuals), followed by high school graduates at 20.4% (30 individuals), individuals with a diploma at 5.4% (8 individuals), elementary school attendees at 4.8% (7 individuals), and one individual (0.7%) holding a doctoral degree. This distribution highlights significant variations in the demographic profiles of pet owners engaging with the VET PROMPT notification system.

Furthermore, the demographic profile of pet owners using the VET PROMPT system predominantly consists of students, who represent a substantial majority at 86.4% (127 individuals). Freelancers and unemployed individuals follow this group, making up 2.7% of the population (4 individuals each). Additionally, the dataset includes 2 company employees and 1 official, accounting for 1.4% and 0.7%, respectively. In terms of salary, a significant proportion of pet owners, 80.3% (118 individuals), reported earning less than $10,000, while those earning between $10,000 and $20,000 constituted 17% (25 individuals). A smaller fraction, 2% (3 individuals), earned between 20,001 and 50,000, while only one individual, making up 0.7% of the sample, reported earning over 50,001. Cats were the most common pet ownership type, owned solely by 52.4% of the respondents (77 individuals); the details show that 27.2% (40 individuals) owned dogs solely, while 20.4% (30 individuals) owned both dogs and cats. This breakdown highlights the diverse socioeconomic statuses and pet ownership preferences among users of the VET PROMPT system. When the pet owner had successfully registered through the system, and an appointment became due, the system immediately sent a notification message through the VET PROMPT system to the pet owner, reminding them to take the animal to the animal hospital. The system then sent a satisfaction questionnaire to those who received the notification promptly.

All 11 questions were mapped as follows:

Q1: Correctness of notifications online

Q2: Appropriateness of notification time

Q3: Usefulness of notifications through online application

Q4: Overall satisfaction with notifications through the online application

Q5: Ease of access to notification information through the online application

Q6: Ease of use for notifications through the online application

Q7: Facilitation of requesting additional information through the online application

Q8: Simplicity of notifications through the online application

Q9: Ease of understanding the notification of the online application

Q10: Ease of understanding the notification of the online application

Q11: Timeliness of receiving veterinarian’s appointment notifications

The analysis of pet owners’ satisfaction with the VET PROMPT system through the Line application revealed a high overall satisfaction, with a mean score of 4.16 and a standard deviation of 0.79. When considering each item, pet owners’ satisfaction with ‘Ease of use for notifications through online application’ was very high (x̄ = 4.25, S.D. = 0.78), and “Simplicity of notifications through online application’ also received a very high rating (x̄ = 4.22, S.D. = 0.76). ‘Correctness of notifications through online application’ achieved a high level of satisfaction (x̄ = 4.03, S.D. = 0.90). Regarding the ‘Appropriateness of notification times,’ satisfaction was high (x̄ = 4.08, S.D. = 0.89). ‘Usefulness of notifications through online application’ was also rated at a high level (x̄ = 4.20, S.D. = 0.80). ‘Overall satisfaction with notifications through online application’ was rated at a high level (x̄ = 4.18, S.D. = 0.75).

Cumulatively, these scores underpin a robust level of satisfaction across various facets of the notification system, highlighting its effectiveness in engaging pet owners effectively.

**Figure 1.**
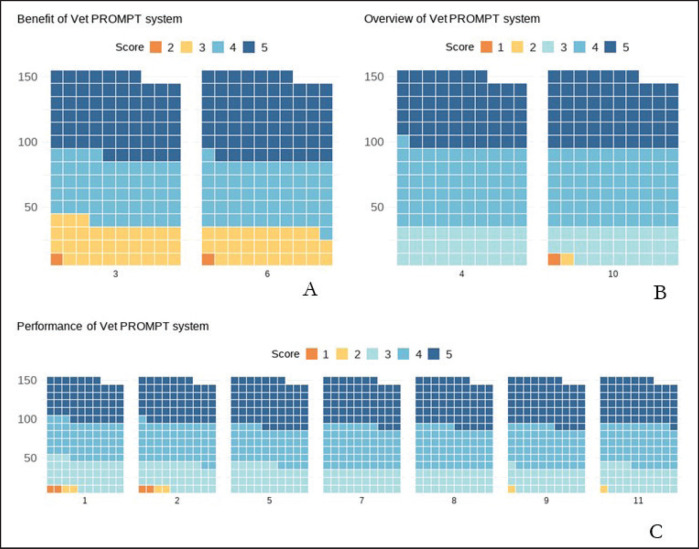
Waffle charts illustrating the perceived benefits of the system based on responses to Questions 3 and 6. (A) The overview of the system based on responses to Questions 4 and 10 (B), The performance of the system based on responses to Questions 1, 2, 5, 7, 8, 9, and 11 (C).

Moreover, ‘Ease of access to notification information through the online application’ had a high satisfaction level (x̄ = 4.18, S.D. = 0.80). ‘Facilitation of requesting additional information through online application’ had a high satisfaction level (x̄ = 4.20, S.D. = 0.76). ‘Timeliness of receiving veterinarian’s appointment notifications’ was rated at a high level (x̄ = 4.16, S.D. = 0.80). ‘Ease of understanding notification of online application’ had a high satisfaction level (x̄ = 4.20, S.D. = 0.78). ‘Overall pattern of notifications through online application’ had a high satisfaction level (x̄ = 4.16, S.D. = 0.80). Overall satisfaction with VET PROMPT was high, with a mean score of 4.16 and a standard deviation of 0.79, indicating a generally positive response, as shown in Table 1. Cronbach’s Alpha of the Benefit of the VET PROMPT system was 0.84, indicating good reliability. Performance of the Benefit of the VET PROMPT system was 0.95, indicating excellent reliability; the overview of the VET PROMPT system was 0.89, indicating good reliability; and the overall reliability of the VET PROMPT system was 0.97, indicating excellent reliability. The benefits of the VET PROMPT system (Questions 3 and 6) are shown in Figure 1A, The overview of the VET PROMPT system (Questions 4 and 10) in Figure 1B, and the performance of the benefits of the VET PROMPT system (Questions 1, 2, 5, 7, 8, 9, and 11) in Figure 1C.

For Question 3, the scores were as follows: 1(0, 0%), 2(1, 0.7%), 3(32, 21.8%), 4(51, 34.7%), 5(63, 42.9%).

**Table 1.** Personal information on pet ownership (*n* = 147).

Items	Level of pet owners’ satisfaction			
		S. D.	Interpretation	Cronbach’s alpha
1. Benefit of the VET PROMPT system				
Usefulness of notifications through an online application	4.20	0.80	High	
Ease of use for notifications through the online application	4.25	0.78	Very High	
2. Performance of the Benefit of the VET PROMPT system				
Correctness of notifications through the online application	4.03	0.90	High	
Appropriateness of notification time	4.08	0.89	High	
Ease of access to notification information through an online application	4.18	0.80	High	
Facilitation of requesting additional information through the online application	4.20	0.76	High	
Simplicity of notifications through the online application	4.22	0.76	Very High	
Ease of understanding the notification of the online application	4.20	0.78	High	
Timeliness of receiving the veterinarian’s appointment notifications	4.16	0.80	High	
3. The overview of the VET PROMPT system				
Overall satisfaction with notifications through the online application	4.18	0.75	High	
Overall pattern of notifications through the online application	4.16	0.80	High	
Total	4.176	0.8079	High	0.97 (Excellence)

For Question 6, the scores were 1(0, 0%), 2(1, 0.7%), 3(28, 19.6%), 4(52, 36.4%), and 5(66, 46.2%).

For Question 1, the scores were as follows: 1(2, 1.4%), 2(2, 1.4%), 3(39, 26.5%), 4(50, 34%), 5(54, 36.7%).

For Question 2, the scores were 1(2, 1.4%), 2(2, 1.4%), 3(34, 23.1%), 4(53, 36.1%), 5(56, 38.1%).

For Question 5, the scores were 1(0, 0%), 2(0, 0%), 3(36, 24.5%), 4(48, 32.7%), 5(63, 42.9%).

For Question 7, the scores were 1(0, 0%), 2(0, 0%), 3(30, 20.4%), 4(57, 38.8%), 5(60, 40.8%).

For Question 8, the scores were 1(0, 0%), 2(1, 0%), 3(30, 20.4%), 4(55, 37.4%), 5(62, 42.2%).

For Question 9, the scores were 1(0, 0%), 2(1, 0.7%), 3(30, 20.4%), 4(55, 37.4%), 5(61, 41.5%).

For Question 11, the scores were 1(0, 0%), 2(1, 0.7%), 3(33, 22.4%), 4(55 37.4%), 5(58, 39.5%).

For Question 4, the scores were 1(0, 0%), 2(0, 0%), 3(30, 20.4%), 4(61, 41.5%), 5(56, 38.1%).

For Question 10, the scores were 1 (1, 0.7%), 2(1, 0.7%), 3(28, 19%), 4(60, 40.8%), 5(57, 38.8%).

**Figure 2.**
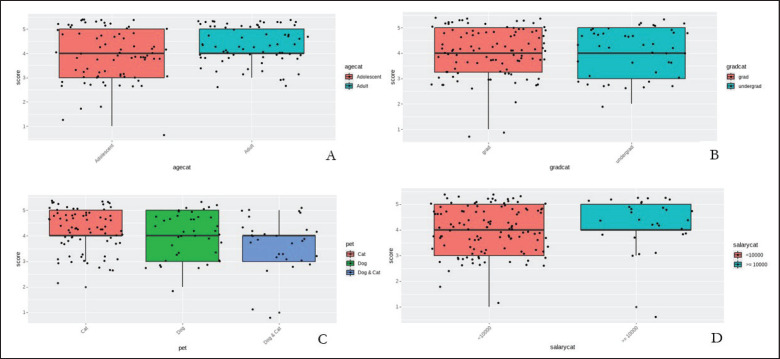
Boxplot showing median, Q1, Q3, and IQR scores of each age category. (A), score of each graduation category. (B), score of each pet category. (C), score of each salary category (D).

According to the analysis, Table 2 shows the effect of pet type, age, education, and salary on the pet owner’s satisfaction score. Dog owners have a chance to rate the application higher than both owners by 1.522-fold, and cat owners have a chance to rate the application higher than both owners by 2.279-fold. Adults seem to be more satisfied with the application than adolescents, with a 1.490-fold chance to rate it higher. Education level didn’t have an effect on the owner’s satisfaction level. Participants with more than 10,000 THB salary have a 1.724-fold chance to rate higher than participants with lower salaries. The boxplot shows the median, Q1, Q3, and IQR score of each age category. Adolescents have a median of 4, Q1 = 3, Q3 = 5, and IQR = 2. Adult has median = 4, Q1 = 4, Q3 = 5, and IQR = 1, as shown in Figure 2A. The graduation category boxplot shows the median, Q1, Q3, and IQR scores of each graduation category. Graduate has median = 4, Q1 = 3.25, Q3 = 5, IQR = 1.75. Undergraduate median = 4, Q1 = 4, Q3 = 5, and IQR = 2, as shown in Figure 2B. The pet category, boxplot shows the median, Q1, Q3, and IQR scores of each pet category. Cat has median = 4, Q1 = 4, Q3 = 5, and IQR = 1. Dog has median = 4, Q1 = 3, Q3 = 5, IQR = 2. Dog & Cat has median = 4, Q1 = 3, Q3 = 4, and IQR = 1 and is represented in Figure 2C, while the salary category boxplot shows the median, Q1, Q3, and IQR scores of each salary category. < 10,000 THB has median = 4, Q1 = 3, Q3 = 5, IQR = 2. ≥ 10,000 THB median = 4, Q1 = 4, Q3 = 5, IQR = 1. (D) is shown in Figure 2D, respectively.

**Table 2.** Comparison of the effect of different factors on the overall pet owner’s satisfaction score.

Factor	OR	95 %CI	*p*–value	Mean ± SD
Both	Reference Value			3.93 ± 0.837
Dog	1.522	1.173 1.976	0.0015*	4.13 ± 0.752
Cat	2.279	1.798 2.891	1.05e–11*	4.28 ± 0.793
Adolescent (< 21)	Reference Value			4.04 ± 0.848
Adult (≥ 21)	1.490	1.218–1.823	0.0001*	4.31 ± 0.722
Bachelor’s degree and above	Reference Value			4.16 ± 0.798
Lower than a Bachelor’s degree	1.132	0.916–1.399	0.25	4.18 ± 0.814
Salary < 10,000 THB	Reference Value			4.10 ± 0.803
Salary ≥ 10,000 THB	1.724	1.337 –2.227	0.00004*	4.43 ± 0.749

*Statistically significant at *p* ≤ 0.05

Based on the above information in Figure 2, the overall scores for each category are shown in Table 3 as follows.

## Discussion

The concept of satisfaction assessment, as an evaluative emotional response toward learning experiences, integrates expectations and psychological needs to effectively pursue and achieve desired outcomes [26]. In a related vein, our study identified that most pet owners were students, comprising 127 individuals (86.4%). This demographic alignment is consistent with findings by Charmaraman et al. [3], who noted that most pet owners, predominantly adolescents aged between 11 and 16 years (average 12.7 years), are inclined towards using social media and online systems for information retrieval, underscoring a preference for digital communication. Furthermore, Lima et al. [27] examined customer satisfaction within pet food subscription services and analyzed 28,786 online reviews from the PET FOOD SOS platform, revealing high satisfaction levels attributed to the clarity of information on product benefits and ingredients, as well as effective advertising visuals. However, they noted that the absence of clear pricing was a critical factor affecting purchasing decisions. Similarly, our findings highlighted that most pet owners appreciated receiving vaccine notifications through the VET PROMPT system, indicating a high satisfaction level. This parallels observations by Sivakumar et al. [28], who reported that accessible notifications and guidelines significantly enhanced self-care practices among patients with heart failure. These findings collectively suggest that tailored, clear, and accessible information delivery enhances user satisfaction across various digital platforms.

**Table 3.** The overall score divided by each category.

Variables	*n*	Minmum	Maximum	Median	Q1	Q3	IQR	Mean	SD
Question 1	147	2	5	4	4	5	1	4.24	0.782
Question 2	147	3	5	4	4	5	1	4.18	0.747
Question 3	147	2	5	4	4	5	1	4.2	0.799
Question 4	147	2	5	4	4	5	1	4.16	0.791
Question 5	147	1	5	4	4	5	1	4.16	0.803
Question 6	147	3	5	4	4	5	1	4.22	0.763
Question 7	147	2	5	4	4	5	1	4.2	0.782
Question 8	147	3	5	4	4	5	1	4.18	0.803
Question 9	147	1	5	4	3	5	2	4.08	0.888
Question 10	147	3	5	4	4	5	1	4.2	0.758
Question 11	147	1	5	4	3	5	2	4.03	0.902
Female	57	1	5	4	4	5	1	4.21	0.769
Male	90	1	5	4	3	5	2	4.14	0.822
Adolescence (18–20 years old)	78	1	5	4	3	5	2	4.04	0.848
Adult (21–60 years old)	69	1	5	4	4	5	1	4.31	0.722
Elementary School	7	3	5	4	3	5	2	4.14	0.854
High school	30	2	5	4	3	5	2	4.13	0.834
Diploma	8	2	5	4.5	4	5	1	4.42	0.656
Bachelor’s degree	101	1	5	4	4	5	1	4.16	0.797
Doctoral’s degree	1	5	5	5	5	5	0	5	0
College students	1397	1	5	4	4	5	1	4.13	0.806
Officialdom	11	4	4	4	4	4	0	4	0
Company employee	22	3	5	4.5	3	5	2	4.14	0.941
ETC	99	3	5	5	4	5	1	4.54	0.66
Freelance	44	4	5	5	4	5	1	4.73	0.451
Unemployed	44	3	5	4.5	3	5	2	4.2	0.878
≤ 10,000	118	1	5	4	3	5	2	4.11	0.803
10,000–20,000	25	1	5	5	4	5	1	4.38	0.776
20,001–50,000	3	4	5	5	4	5	1	4.61	0.496
> 50,001	1	5	5	5	5	5	0	5	0
Dog	40	2	5	4	4	5	1	4.13	0.752
Cat	77	1	5	4	4	5	1	4.28	0.793
Dog and Cat	30	1	5	4	3	5	2	3.93	0.837

Contemporary animal hospitals are increasingly adopting online applications to enhance their service delivery, as evidenced by the development of the CARE A PET application. This app, available on the Android system, is tailored to meet the specific needs of pet owners, enabling them to locate nearby animal hospitals and access both regular and emergency veterinary services. Pet owners can input information or directly contact a veterinarian through the app, significantly alleviating the challenges of transporting pets for treatment, especially in rural areas [29]. Similarly, the VET PROMPT system has improved user satisfaction by simplifying the process for pet owners to request additional information. During the COVID-19 pandemic, travel and treatment difficulties prompted the adoption of the TerraVet system, which utilizes both smartphone and web applications for communication between pet owners and veterinary clinics. This integration has garnered positive feedback, with users advocating for an added payment feature to facilitate comprehensive services and enable round-the-clock communication with veterinary hospitals from home [30]. This trend reflects a broader movement towards digital solutions that streamline interactions between pet owners and healthcare providers, enhancing accessibility and satisfaction.

Pet owners have expressed high satisfaction with the ease of accessing veterinary information through online applications, appreciating the simplicity of receiving notifications and the facilitated communication with veterinarians. This positive reception aligns with the findings of Coe et al. [31], who conducted a focus group study involving 24 veterinarians and 32 pet owners, uncovering communication challenges in clinics, such as the provision of critical, yet often incorrect or incomplete, information during constrained interactions. Correspondingly, Shaw et al. [32] investigated communication patterns in veterinary practices by observing 50 companion animal practitioners and 300 pet owners in southern Ontario. Their study, which examined notifications during six clinical appointments—three for wellness and three for health issues—revealed that notifications were provided in two forms: Biomedical (58%) and biolifestyle social communication (42%). These forms varied depending on the appointment type and duration, affecting the relationship with appointment notifications. Their findings suggest that online communication platforms can mitigate time limitations and enhance the consultation experience, aligning with pet owners’ preferences for more extended and thorough interactions with their veterinarians.

Pet owners have also reported high levels of satisfaction with the completeness, correctness, and usefulness of the veterinary information provided through online applications. This satisfaction aligns with the findings of Nwabueze and Oju [33], who demonstrated that digital applications facilitating doctor–patient interactions are highly effective in delivering continuously updated information. As well, Ameta et al. [34] conducted a study on a medication reminder and healthcare Android application, and 100% of the users reported that the health information received through the application significantly benefited their self-care practices and enhanced their health awareness. These studies collectively indicate the effectiveness of digital tools in improving the accessibility and quality of healthcare information, which is also reflected in the veterinary context, where online applications have proven essential for ensuring comprehensive and beneficial health communication between veterinarians and pet owners.

Furthermore, pet owners have conveyed high satisfaction with the ease of use and simplicity of notifications provided by the VET PROMPT online application. This finding aligns with the research conducted by Blancaflor et al. [35], who examined the VETGO system, a mobile application designed to facilitate veterinary services for pets. Their study revealed that 80 % of users in the Philippines were satisfied with the application’s capability to deliver pet medicine and medical services. Furthermore, 60% of the customers positively reviewed the convenience of receiving medical services directly at home. These results highlight a general preference among users for receiving in-home medical delivery services, underscoring the value of mobile applications in enhancing the accessibility and user-friendliness of veterinary care. This convergence of technology and healthcare delivery continues to transform user experiences by providing more flexible and responsive service options. Pet owners articulated high satisfaction with the timeliness of receiving appointment notifications from veterinarians, praising the prompt and updated delivery of information. This communication efficacy significantly contributed to their overall satisfaction and aligns with findings from Regan et al. [36], who observed that the MAS notification system notably improved vaccination uptake and coverage among pregnant women by ensuring they received timely and accurate reminders compared to traditional self-reminder methods. Relatedly, Kempe et al. [37] demonstrated that reminder-recall systems employing automated messages and postcards were more effective in encouraging patients to pursue immunizations than methods involving letters, emails, or phone calls. These studies jointly emphasize the importance of timely and efficient notification systems in enhancing compliance with healthcare schedules and treatments, highlighting their role in improving health outcomes across different patient groups.

The VET PROMPT system has demonstrated high overall satisfaction among users, particularly with the pattern and effectiveness of notifications delivered through the online application. This positive reception echoes the findings of Soleh et al. [38], who evaluated the OPet application designed for online pet shop services such as sales, grooming, daycare, and the procurement of pet supplies in Tangerang City and its districts. Their research indicated that a majority of the 50 participants were highly satisfied with the convenience and functionality of the online services provided. Additionally, satisfaction extends to innovative applications like the PetCare system, as studied by Luayon et al. [39], which offers smart pet care solutions through an IoT mobile application. This system enhances pet management by incorporating features such as automated food and water dispensing and temperature control of pet environments, all operable via a mobile phone.

Table 4 provides a comparative analysis of various online pet healthcare platforms, evaluating features such as type of consultation, Subscription, emergency fund, communication methods, and unique functionalities. Platforms like CareForPaws, Veterinary Online Appointment System (VOAS), and Terravet offer video consultations and comprehensive record access, while OPet Application and VETGO distinguish themselves with real-time booking and responsive teleconsultation. Simpler platforms such as Care a Pet and PET FOOD SOS focus on ease of use and targeted services like SMS reminders and emergency food delivery. Today, pet owners have the opportunity to access veterinary consultations through a range of platforms, including Pawp, WhiskerDocs, Chewy, Dutch, Airvet, Vetster, AskVet, and PetCoach. A comparative evaluation of these services enables pet owners to identify the platform that most effectively aligns with their needs in terms of convenience, communication, and comprehensive veterinary care.

**Table 4.** Pet online healthcare platform comparison.

Platform	Type	Subscription	Emergency fund	Notifications (Email/SMS)	On–demand consults	Reference
CareForPaws	Chat and video chat	No data	No	Both	For member/non–members	[40]
Terravet	Chat	No data	No	Email	For member/non–members	[30]
Care a Pet	Chat	No data	No	SMS	For member/non–members	[29]
PET FOOD SOS	Chat	No data	Emergency Food Delivery	SMS	For member/non–members	[27]
VETGO	Chat	No data	Urgent Care Available	Both	For member/non–members	[35]
OPet	Chat	No data	Some Support	Both	For member/non–members	[38]
VOAS	Chat	No data	No	Both	For member/non–members	[41]
Pawp	Chat and video chat	$99/year up	Yes	No data	For members only	[42]
WhiskerDocs	Chat, phone, text messaging, email, and video	$129.99/year	No	No data	For non–members, $39.99 for a live chat or phone call or $4.99 for an email	[43]
Chewy	Video call and chat	No	No	No data	Free live chats for all Autoship customers in most states. Otherwise, $19.99/video call.	[44]
Dutch	Messaging through your account and video chat	Plans start at $11/month or $132/year	No	No data	For members only	[45]
Airvet	Video call and chat available	$35/month or $350/year	No	No data	For non–members, $75 per video call	[46]
Vetster	Video call and chat available	$19.99/month for a yearly subscription or $24.99 billed every 3 months	No	No data	For non–members, $55 per appointment	[47]
AskVet	Chat	$9.99/month	No	No data	For monthly subscribers only	[48]
PetCoach	Live chat and expert forum	$20 live chat consult, $5 to submit a question to an expert forum	No	No data	$20 live chat consult, $5 to submit a question to an expert forum	[49]

These findings suggest a strong user approval for digital solutions that streamline pet care and management, underscoring the effectiveness of technology-enhanced services in meeting the needs and expectations of pet owners.

## Conclusion

Conclusions derived from utilizing the VET PROMPT system, integrated with the LAOA, show that pet owners are highly satisfied with the notification service provided through the online application. The evaluation revealed strong satisfaction with several aspects of the system: the ease of use and simplicity of the notifications, the completeness, correctness, and usefulness of the veterinary information, and the overall promptness in receiving veterinarians’ appointment notifications. Pet owners particularly valued the timely delivery of notifications, their appropriate timing, and the ease of requesting additional information. The system’s overall architecture, which supports seamless interactions between pet owners and veterinary services, also received positive feedback, particularly for its consistent and efficient pattern of notifications. This online notification system has proven to be an effective tool for animal hospitals, clinics, and veterinarians to swiftly and efficiently communicate crucial information related to pet health and disease prevention. The findings of this study highlight the efficacy of the VET PROMPT system in enhancing communication between veterinarians and pet owners, underscoring the system’s potential to improve pet healthcare management through timely and accurate notifications. However, the study’s limitations include its reliance on self-reported data, which may introduce bias, and the specific focus on users of the LINE application, which may not represent the broader population of pet owners. Future research should investigate the applicability of similar notification systems across different platforms and geographic locations to validate the findings and extend their generalizability. Furthermore, we could conduct comparative studies to evaluate the efficacy of different notification formats and technologies in enhancing pet health outcomes. Further investigation into the long-term impacts of such systems on pet health management practices would also provide deeper insights into their practical benefits and areas for enhancement.

## List of abbreviations

LAOA, LINE Application Official Account; RV, rabies virus; IoT, Internet of Things; NPCC, Novel Pet Collaborative Care; IOC, Index of Congruence; ANOVA, Analysis of variance; VGG, Vaccination Guidelines Group; ICH–GCP, International Conference on Harmonization in Good Clinical Practice; IQR, Interquartile Range; VOAS, Veterinary Online Appointment System.
